# Spatial-temporal data-augmentation-based functional brain network analysis for brain disorders identification

**DOI:** 10.3389/fnins.2023.1194190

**Published:** 2023-05-17

**Authors:** Qinghua Liu, Yangyang Zhang, Lingyun Guo, ZhengXia Wang

**Affiliations:** School of Computer Science and Technology, Hainan University, Haikou, China

**Keywords:** rs-fMRI, spatial-temporal information, functional brain network, data augmentation, brain disorders

## Abstract

**Introduction:**

Due to the lack of devices and the difficulty of gathering patients, the small sample size is one of the most challenging problems in functional brain network (FBN) analysis. Previous studies have attempted to solve this problem of sample limitation through data augmentation methods, such as sample transformation and noise addition. However, these methods ignore the unique spatial-temporal information of functional magnetic resonance imaging (fMRI) data, which is essential for FBN analysis.

**Methods:**

To address this issue, we propose a spatial-temporal data-augmentation-based classification (STDAC) scheme that can fuse the spatial-temporal information, increase the samples, while improving the classification performance. Firstly, we propose a spatial augmentation module utilizing the spatial prior knowledge, which was ignored by previous augmentation methods. Secondly, we design a temporal augmentation module by random discontinuous sampling period, which can generate more samples than former approaches. Finally, a tensor fusion method is used to combine the features from the above two modules, which can make efficient use of spatial-temporal information of fMRI simultaneously. Besides, we apply our scheme to different types of classifiers to verify the generalization performance. To evaluate the effectiveness of our proposed scheme, we conduct extensive experiments on the Alzheimer's Disease Neuroimaging Initiative (ADNI) dataset and REST-meta-MDD Project (MDD) dataset.

**Results:**

Experimental results show that the proposed scheme achieves superior classification accuracy (ADNI: 82.942%, MDD: 63.406%) and feature interpretation on the benchmark datasets.

**Discussion:**

The proposed STDAC scheme, utilizing both spatial and temporal information, can generate more diverse samples than former augmentation methods for brain disorder classification and analysis.

## 1. Introduction

Functional brain network (FBN) analysis, based on resting-state functional magnetic resonance imaging (rs-fMRI), has made positive contributions to the diagnosis of brain diseases and the revelation of the principle of brain diseases (Smith, 2012). As an effective technique to analyze FBNs, machine learning has become a current focus of research by automatically analyzing rs-fMRI data to obtain rules and applying these rules to predict new data (Taschereau-Dumouchel et al., 2022). However, due to the difficulty of acquisition and collection, the sample size of rs-fMRI is scarce compared to classical dataset used in machine learning (Tanveer et al., 2020; Zhang et al., 2023), which may result in under- or over-fitting of the model for FBN analysis (Marek et al., 2022).

To solve the problem of small training sizes, data augmentation has shown great potential in FBN analysis. Numerous studies have directly transferred image data augmentation methods to rs-fMRI data. For example, noise addition (Yang et al., 2020), which is a classical and simple augmentation method for image data, was applied to add different kinds of noise to rs-fMRI data for increasing sample size. However, this method is more geared toward improving the ability of machine learning to resist noise interference, rather than obtaining more diverse samples (Fang et al., 2022). Therefore, it is an important purpose to generate more diversified samples in data augmentation.

Many studies have focused on using efficient algorithms to obtain more diverse samples. For example, Eslami and Saeed (2019) used the extended SMOTE algorithm to generate new samples by linear interpolating different samples in the same category. Yao and Lu (2019) proposed an improved generative adversarial network to augment rs-fMRI functional connectivity data for classification task. Although sample generation based on the above algorithms has achieved good performance in the field of rs-fMRI data augmentation, the speciality (e.g., spatial-temporal information; Yan et al., 2022) of rs-fMRI data that provides valuable information for understanding the pathological mechanism of brain disorders is ignored in these methods.

Considering the importance of temporal information in FBN analysis, more and more studies focus on data augmentation based on time series of rs-fMRI. For example, Dvornek et al. (2017) proposed a data augmentation method by randomly cropping sequences from a time series, which increased the size of the dataset by a factor of 10. Zhu et al. (2021) used random window resampling for data augmentation, which generated more samples by obtaining random consecutive time series from the original brain signal. In addition, Mao et al. (2019) introduced a data augmentation method by sampling fMRI scans into short pieces and taking the sampled pieces as inputs for classification. Qiang et al. (2021) constructed a deep recurrent variational auto-encoder that combined variational auto-encoder and recurrent neural network to aim the small sample size problem of fMRI data. These methods took full account of the temporal information of rs-fMRI data. However, these data augmentation methods ignored the spatial information that is an important structural characteristics of FBN.

To solve the problems of the above mentioned, we propose a novel classification scheme based on spatial-temporal data augmentation that consists of three modules, including spatial augmentation module, temporal augmentation module, and spatial-temporal fusion module. Inspired by jackknife cross-validation, which is effective in reducing linear model bias, we improve the previous sampling method that only used sliding window through discontinuous sampling. On the other hand, as there is often a correlation between adjacent brain regions, using spatial prior information obtained from brain anatomy will help the classifier's analytical ability. Compared with previous approaches, our scheme can produce more diverse training samples and make full use of spatial-temporal information of fMRI. Specifically,

(1) We proposed a spatial data augment method, in which spatial prior knowledge of brain regions is used by a *k*NN-like approach.
(2) Different from previous methods, we randomly extract discontinuous time series form original time period and recombine them. It can generate more diverse training samples than previous methods which only use continuous series.
(3) To prevent the mutual interference of different augmentation rule, we use a tensor fusion method to fuse results of classification after different kinds of augmentation. In this way, we can take spatial-temporal information into account at the same time, which is ignored by previous data-augmentation-based methods.

We validate our proposed scheme mainly on the public ADNI dataset, and the experimental results demonstrate its superiority over other methods. Besides, we train our scheme based on different classifiers (e.g., neural network, random forest, and support vector machine) and compare their effects. Experiments show that our scheme can fit well with different classifiers.

The rest of the paper is organized as follows. During Section 2, we present our scheme and introduce the dataset used in our scheme. The experiments that we take will be introduced during Section 3. Then the comparison between different data augmentation methods and the disease-related features (functional connections) will be discussed in Section 4. Finally, we will summarize the conclusions of this paper in Section 5.

## 2. Materials and methods

In this section, we first introduce the datasets involved in our experiments, including data acquisition and pre-processing. Then we describe the overall pipeline of our proposed scheme in detail.

### 2.1. Experimental datasets

To validate the effectiveness of the proposed method, we perform two datasets for disease classification, including the publicly available ADNI dataset and the MDD dataset.

#### 2.1.1. ADNI dataset

Our main experimental data is from the Alzheimer's disease neuroimaging initiative (ADNI) dataset (Jack et al., 2008). This dataset includes 563 subjects, 154 of whom are normal cognition (NC), 165 of whom are early mild cognitive impairment (eMCI), 145 of whom are late mild cognitive impairment (lMCI), and 99 of whom are Alzheimer's disease (AD). The acquisition parameters are as follows: in-plane image resolution = 2.29–3.31 mm, slice thickness = 3.31 mm, echo time (TE) = 30 ms, repetition time (TR) = 2.2–3.1 s, and the scanning time for each subject is 7 min (resulting in 140 volumes).

#### 2.1.2. MDD dataset

To demonstrate the effectiveness of our scheme on different rs-fMRI datasets, a major depressive disorder (MDD) dataset provided and pre-processed by the REST-meta-MDD project (Yan et al., 2019; Chen et al., 2022) is experimented in Section 3. The REST-meta-MDD dataset consists of 1,276 major depressive disorder samples from 24 different sites. Specifically, patients with major depressive disorder (MDD) contain 463 men and 813 women, normal controls (NC) contain 462 men and 641 women. The acquisition parameters are as follows: repetition time (TR) = 2,000–3,000 ms; echo time (TE) = 25–40 ms; flip angle = 30 or 90°; slice number = 22–39.

### 2.2. Data preprocessing

The pipeline of pre-processing for MDD dataset is provided by the REST-meta-MDD project, which consists of head motion correction, spatial normalization, non-linear registration, and spatial smoothing, and so on (Yan et al., 2019).

However, since we only have access to the preprocessed MMD dataset, it is difficult to control the preprocessing pipeline. Therefore, for the sake of fairness, we employ a similar pipeline as in the MDD dataset to pre-process the ADNI dataset in our experiments. We use the Data Processing Assistant for Resting-State fMRI (DPABI) toolbox (Yan et al., 2016) to preprocess the fMRI data.

Specifically, we discard the initial 10 volumes to avoid outliers and performed slice-timing correction. After that, the time series of images for each subject are realigned using a six-parameter (rigid body) linear transformation. Then, we co-register individual T1-weighted images to the mean functional image using a 6 degrees-of-freedom linear transformation without re-sampling. The Friston 24-parameter model is utilized to regress out head motion effects. Finally, we map the fMRI data to the 116 brain regions of the AAL template, convert them into time-series signals, and normalize these signals (Friston et al., 2000). For each subject, we can get 137 time points for each region of interest (ROI) of AAL template after above steps.

### 2.3. Methods

**Figure 1:**
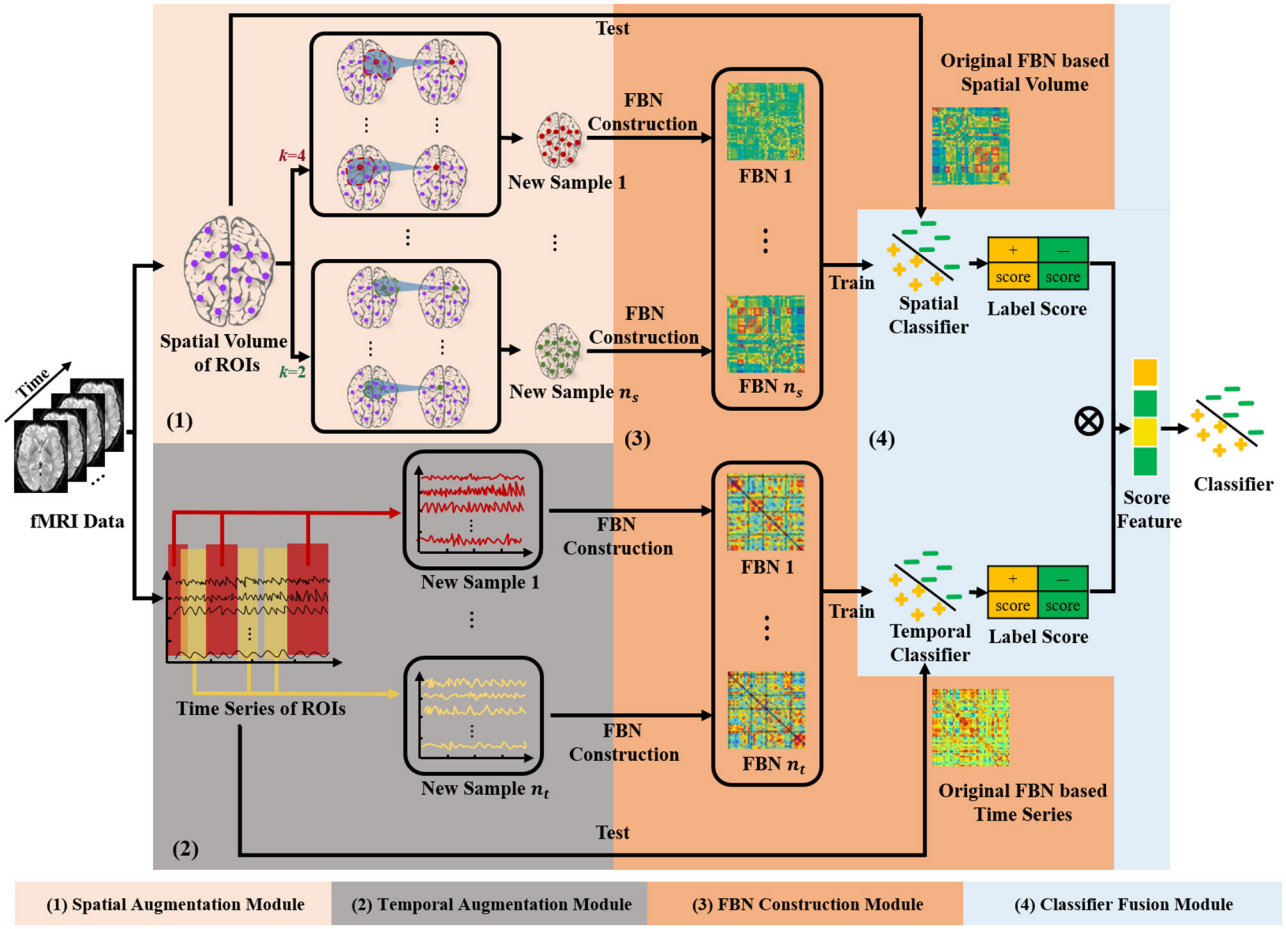
Illustration of the proposed scheme based on spatial-temporal data augmentation (STDAC).

The framework of our STDAC scheme is shown in Figure 1, which consists of three modules, including spatial augmentation module, temporal augmentation module, and spatial-temporal fusion module. We denote $X=\left[x_{1};x_{2};\;\cdots \;;x_{m}\right]\in R^{t\times m}$ as the matrix of rs-fMRI data, where *m* is the total number of nodes, *t* is the number of time points, $x_{i}={\left[x_{i}^{1},x_{i}^{2},\;\cdots \;,x_{i}^{t}\right]}^{T}$ is the time series of the *ith* (*i* = 1, ⋯ , *m*) node.

#### 2.3.1. Spatial augmentation module

Previous studies have found that brain regions with similar spatial information have similar representations, and various methods for FBN analysis have been proposed based on the spatial information of fMRI (Zou et al., 2022). For example, many FBN division methods (Power et al., 2011; Ji et al., 2019) based on spatial feature have been proposed to search the discriminative subnetworks that are significant for brain disease (Sheline and Raichle, 2013). In our proposed STDAC scheme, we use a *k*NN-like approach to augment samples with spatial prior knowledge of brain regions.

As shown in the module (1) of Figure 1, we first measure the Euclidean space distance between node *i* and other nodes based on the AAL template. The measurement method is provided by the nilearn library (Abraham et al., 2014). Then we update the time series *x*_*i*_ of node *i* based on the *k* nearest neighbors (nodes) to obtain the new time series *y*_*i*_. Specifically, we put the *k* nearest neighbors of node *i* in a set *C*_*i*_ which has the following restrictions:


(1) $$\begin{array}{l} \{\begin{array}{cc} |C_{i}|\leq k+1\\ d\left(i,s\right)<d\left(i,j\right) & \left(s\in C_{i},j\notin C_{i}\right)\\ d\left(i,s\right)<r*D & \left(s\in C_{i},0<r<1\right) \end{array} \end{array}$$

where *d*(·) is the Euclidean space distance, *r* is a constant number, and *D* is the biggest distance in AAL template. Finally, the node *i* can be updated by the following equation:


(2) $$\begin{array}{l} y_{i}=mean\left(C_{i}\right). \end{array}$$

After all nodes of the brain are updated, we can get a new sample (brain). Note, we can obtain different samples by adjusting the size of *k*. Once we obtain the new sample, we utilize Pearson's correlation (PC) to estimate the FBN, which can capture the full correlation between nodes and has gradually become one of the benchmark methods in this field. With the increase of training data, the ability of the model to use high-dimensional features is also improved. So we did not perform feature selection commonly used in conventional FBN analysis methods.

#### 2.3.2. Temporal augmentation module

Temporal information in rs-fMRI is essential for FBN analysis and is commonly employed in data augmentation studies. Previous methods usually use time windows to generate more samples by obtaining random consecutive time series from raw brain signals. Unlike employing consecutive time series, the time period obtained can be random in our scheme, meaning that it can generate more training samples from a single original sample.

Specifically, we obtain new time series *z*_*i*_ by randomly selecting *x*_*i*_ and reorganizing the selected vectors as the following equation:


(3) $$\begin{array}{l} z_{i}=\left[\begin{array}{c} z_{i}^{1}\\ z_{i}^{2}\\ \cdots \\ z_{i}^{l} \end{array}\right]\in R^{l}\;\left(z_{i}^{1},\cdots \;,z_{i}^{l}\in random\left\{x_{i}^{1},\cdots \;,x_{i}^{t}\right\},l<t\right) \end{array}$$

where *l* denotes the number of selected time points, which should be smaller than *t*. We limit the minimum number of sampling points to prevent too few samples, which may cause the sampling position to be too sparse. Jackknife cross-validation is a method that excels at reducing errors in linear regression models and is widely used in statistical analysis. According to jackknife cross-validation, this method of randomly selecting time periods without caring about continuity would be helpful to obtain an average value when computing the Pearson correlation (Barber et al., 2021), which calculates the linear correlation between two sets of data. We try to use this method to reduce estimation bias of brain interval connections. Similar to the spatial augmentation module, we then construct FBN based on these augmented samples.

#### 2.3.3. Spatial-temporal fusion module

In this spatial-temporal fusion module, we fuse the results extracted from temporal and spatial data augmentation module to prevent the mutual interference of samples generated by different augmentation rules. Different from feature fusion, we perform the tensor fusion method to fuse the results of classification after temporal data augmentation and spatial data augmentation.

**Figure 2:**
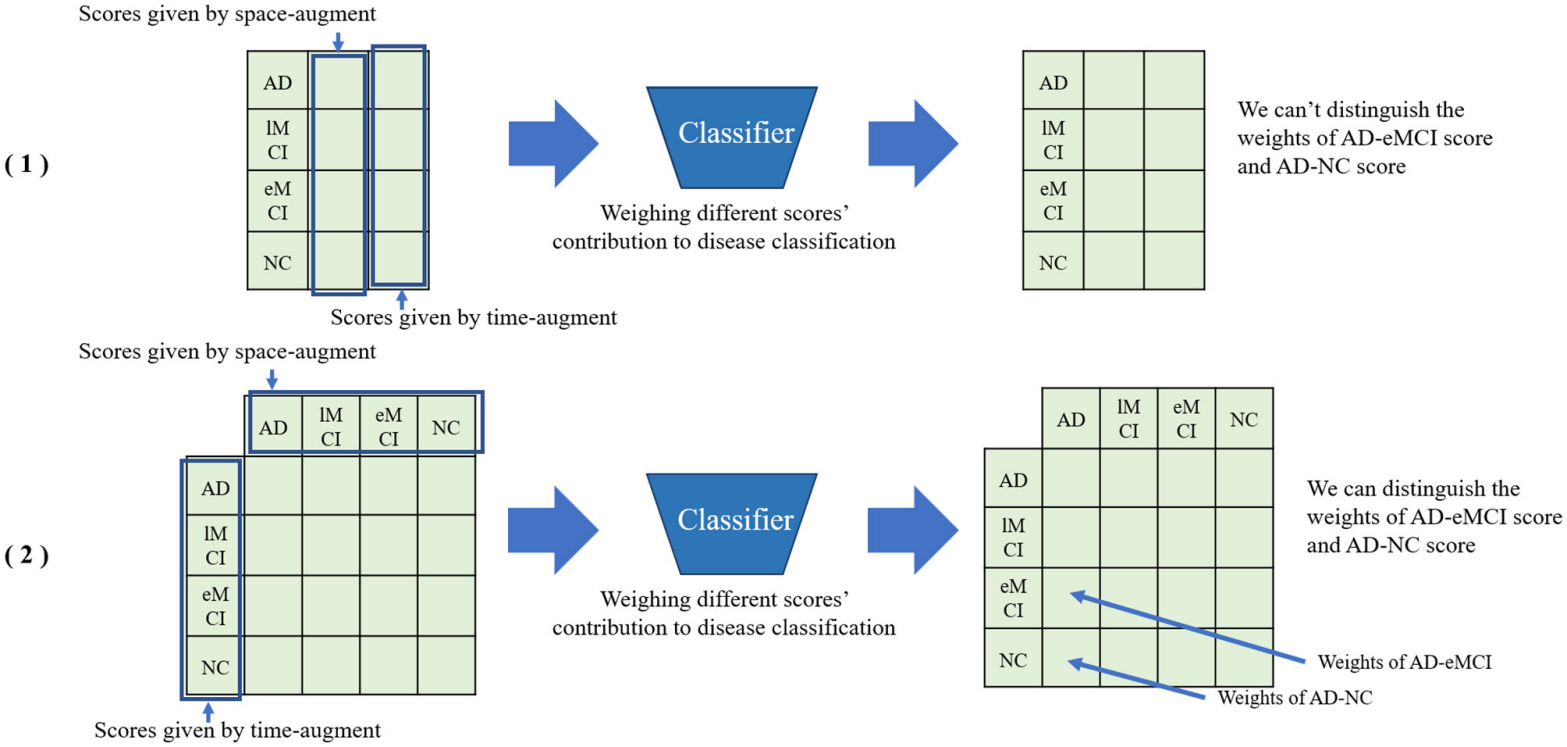
Comparison between directly concatenating method and matrix multiplication method for feature fusion. (1) Directly concatenating method. (2) Matrix multiplication method.

As shown in the module (4) in Figure 1, we train two classifiers with the augmented samples based on different modules, and obtain the classification results by the original sample as a test. Specifically, we use multiplication operations on the high-dimensional maps of the two outcome tensors. Specifically, we transpose the vector of label score obtained from spatial augmentation and multiply it with the temporal score vector. Compared to directly concatenating two vectors, multiplying them can better preserve the potential information in the vectors and assist the classifier in assigning weights to the fused vectors. As shown in Figure 2, directly concatenating method will lose some potential score information for further classification. The fused score feature ω is calculated by the following equation:


(4) $$\begin{array}{l} \omega =f\left({S_{spatial}}^{T}S_{temporal}\right) \end{array}$$

where *S* denotes the label score extracted by two augmentation modules, *f*(·) is a de-averaging and normalizing function commonly used in machine learning. Finally, the label score ω obtained after fusion will be delivered to the classifier of the spatial-temporal fusion module.

## 3. Experiments and results

In this section, we first introduce the four comparison methods in the following experiments. we then present the implementation details, parameter settings of our proposed STDAC scheme, and the valuation metrics for the classification task. And then we report the experimental results of the comparison methods in ADNI dataset. Finally, the effectiveness of our proposed scheme on different datasets is demonstrated convincingly.

### 3.1. Comparison methods

**Figure 3:**
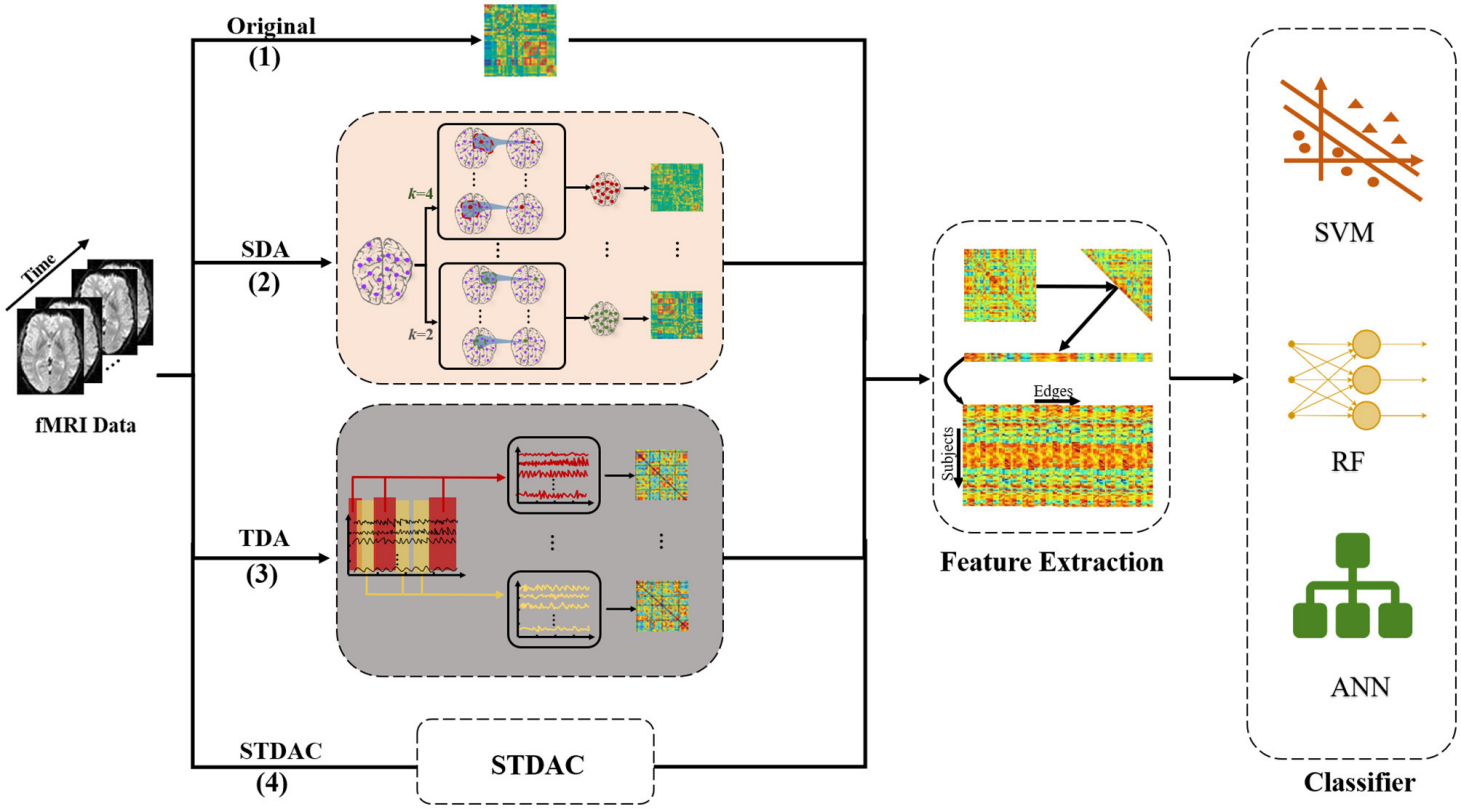
Different schemes for comparison. (1) **Original**, a scheme that does not use data augmentation, (2) **SDA**, a scheme only with spatial data augmentation module, (3) **TDA**, a scheme only with temporal data augmentation module, and (4) **STDAC**, our proposed scheme.

As shown in the Figure 3, we compare our proposed STDAC with three methods, including (1) **Original**, a scheme that does not use data augmentation, (2) **SDA**, a scheme only with spatial data augmentation module, (3) **TDA**, a scheme only with temporal data augmentation module, and (4) **STDAC**, our scheme. Besides, three popular classifiers are employed to test the effectiveness of our scheme, including support vector machine (SVM), random forest (RF), and artificial neural networks (ANN).

### 3.2. Implementation details and evaluation metrics

Our STDAC scheme is an offline mode, where the training phase is separated from the testing phase. The training phase shown in Figure 1 has been described in detail in Section 2.3. In our experiments, for the spatial augmentation module, we generate four times more training samples based on the spatial prior knowledge. And for the temporal augmentation module, we generate 100 times more training samples based on the time series. After the training phase, the parameters of the classifier in the test phase are inherited from the classifier in the training phase. We then use the learned classifier to classify the test data.

To obtain an unbiased evaluation, all experiments employ a 10-fold cross-validation method. Specifically, the dataset is randomly divided into ten equal-sized subsets, nine of which are selected for training and the remaining 1 is used for testing. We repeat the whole process 10 times and finally average these 10 results. To prevent learning samples generated from test data during model training, which may result in erroneous experimental results, we divide the data into test dataset and training dataset before the data augmentation stage and only did data augment for the training dataset, not for the test dataset. Besides, we use three indicators (i.e., *Accuracy*, *Precision*, and *Recall*) as performance evaluation metrics, which are commonly used in machine learning classifiers.

### 3.3. Classification results

**Figure 4:**
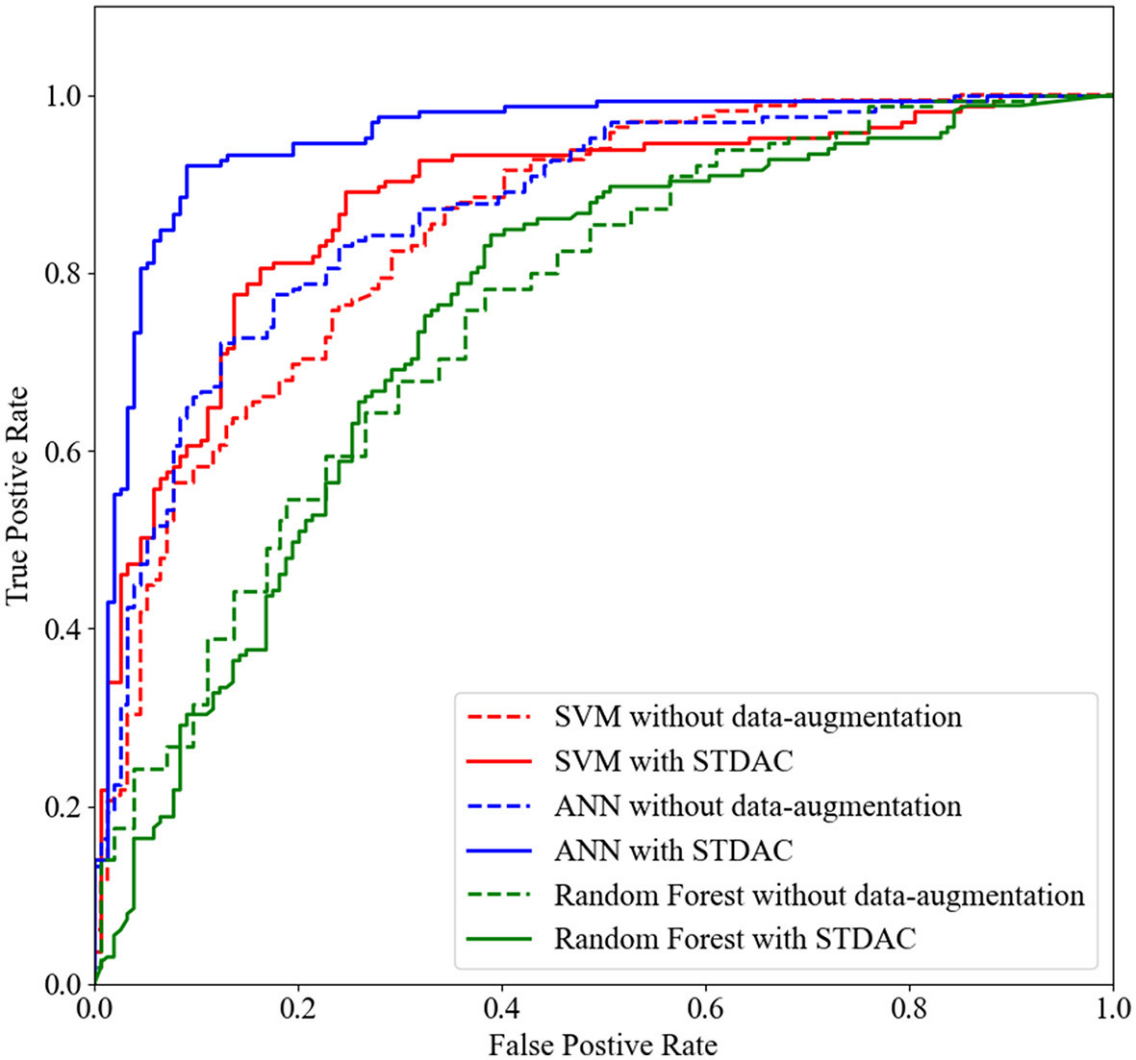
The ROC curves achieved by original method and STDAC scheme in the task of NC vs. eMCI classification.

Table 1 summarizes the results of three methods with different classifiers in the four-class classification task, and Figure 4 plots the corresponding ROC curves. From Table 1 and Figure 4, we have the following interesting observations.

(1) As shown in Table 1, our spatial-temporal augmentation scheme can achieve better results in most cases. It shows that our STDAC scheme based on data augmentation is effective in reducing over fitting or under fitting of classification model. Moreover, the improvement in classifier performance with spatial-temporal augmentation (STDAC) is often better than that with data augmentation using only one augmentation module (SDA and TDA), which verifies the necessity and effectiveness of our proposed spatial-temporal fusion module.
(2) Among all competing classification algorithms, the performance of temporal augmentation module is better than that of spatial augmentation module, which may be resulted by the number of generated samples. In our scheme, the spatial augmentation module generates 4-fold samples, while the temporal augmentation module can generate 100-fold samples. Besides, we discuss the effect of different augmentation degrees in Section 4.3.
(3) We draw ROC curves using our scheme and original method with different classifiers in Figure 4. It can be observed that our scheme fits well with ANN in particular, which may be related to the fact that neural networks are better suited for processing large amounts of data.

**Table 1:** Classification results of three methods with different classifiers in the four-class classification task.

**Classifier**	**Augment method**	**Accuracy**	**Precision**				**Recall**			
			**NC**	**EMCL**	**LMCL**	**AD**	**NC**	**EMCL**	**LMCL**	**AD**
SVM	Original	63.772	61.042	75.147	59.333	55.667	70.363	57.987	60.432	81.651
	SDA	73.387	70.083	81.213	69.381	70.778	70.790	69.984	74.587	85.532
	TDA	78.706	74.625	84.265	**76.429**	**78.889**	**78.217**	**74.641**	79.518	90.944
	STDA	**79.054**	**79.125**	**84.853**	73.619	76.889	73.980	74.012	**84.226**	**94.167**
RF	Original	51.513	57.792	65.515	45.571	27.222	51.362	48.143	53.403	79.833
	SDA	53.828	56.458	69.191	46.095	35.333	50.480	48.879	**61.134**	81.000
	TDA	60.576	**65.042**	**74.007**	52.952	42.556	60.195	57.968	59.396	**84.369**
	STDA	**61.830**	59.833	69.118	**58.476**	**57.667**	**66.814**	**62.485**	57.421	63.566
NNs	Original	65.909	64.917	62.426	59.238	83.000	71.501	66.164	66.017	62.749
	SDA	70.338	68.792	64.669	75.095	74.556	75.040	73.741	65.351	75.399
	TDA	82.218	80.458	**84.596**	78.762	**86.000**	**82.849**	81.066	**84.097**	87.965
	STDA	**84.170**	**85.450**	83.770	**86.367**	83.934	81.083	**86.544**	80.714	**90.000**

The bold values highlight the best performances in experiments.

### 3.4. Experiments in other datasets

To further verify the performance of our SDTA scheme, we employ a MDD dataset for the eMCI vs. NC classification task. The experimental results are reported in Table 2. It can be observed that our scheme achieves the best performance in the ablation experiments, which indicates the good robustness of our proposed model. It has been confirmed that data augmentation using spatial-temporal information can help boost classification performance for brain diseases. Besides, the effect of our model is weaker in the MDD dataset than that in the ADNI dataset. The one possible reason is the structural changes in the brain are stronger in neurological diseases (e.g., AD) than in psychiatric diseases (e.g., MDD) (Yan et al., 2019). Another reason is that the REST-meta-MDD dataset comes from 24 different sites, where different scanning devices and environments at different sites can lead to accuracy degradation of the classification model.

**Table 2:** Classification performance in the task of MDD vs. NC classification.

	**Accuracy**	***F*_1_-score**	**Precision**		**Recall**	
			**NC**	**MDD**	**NC**	**MDD**
Original	60.060	57.913	60.829	59.191	62.908	57.087
SDA	62.219	59.145	63.219	61.091	64.839	59.541
TDA	62.305	60.437	63.455	60.996	64.971	59.451
STDAC	**63.406**	**61.325**	**64.168**	**62.527**	**66.134**	**60.569**

The bold values highlight the best performances in experiments.

## 4. Discussion

In this section, we discuss the performance comparison between our scheme and previous related methods, the weights of the ROI obtained by our modality, the comparison of our scheme in different augmentation degrees, as well as the drawbacks and future of our work.

### 4.1. Experiments of comparison with related methods

We compare our model with the latest data augmentation methods in the field of FBN analysis, including the random window-based data augmentation (Zhu et al., 2021), improved SMOTE (Eslami and Saeed, 2019), auto-encoder-based generative model (Ohno, 2020), conditional generative adversarial network (Raja and Kannimuthu, 2023), and GANSO (Salazar et al., 2021), with experimental results reported in Table 3.

**Table 3:** Classification performance of the related methods for comparison.

	**Accuracy**	**Precision**				**Recall**			
		**NC**	**eMCI**	**lMCI**	**AD**	**NC**	**eMCI**	**lMCI**	**AD**
The random window-based data augmentation	76.046	70.708	81.801	74.952	75.667	72.661	72.894	77.829	87.433
Improved SMOTE	73.189	74.625	**84.890**	69.619	56.556	69.305	68.965	76.500	**95.655**
Auto-encoder-based generative model	65.360	61.625	78.199	60.714	56.444	69.437	60.680	62.377	81.357
Conditional generative adversarial network	73.061	77.780	74.281	71.281	73.701	69.750	65.772	**81.238**	77.667
GANSO	79.189	74.107	72.398	**87.547**	**95.714**	79.042	85.993	73.857	75.778
STDAC (our method)	**84.170**	**85.450**	83.770	86.367	83.934	**81.083**	**86.544**	80.714	90.000

The bold values highlight the best performances in experiments.

Compared with previous data augmentation methods, our STDAC scheme achieves the best performance on the classification task in the ADNI dataset. Different from the random window-based data augmentation, which uses time windows to generate more samples by obtaining random consecutive time series, our scheme of randomly selecting time periods without caring about continuity would be helpful to obtain an average value when computing the Pearson matrix (Barber et al., 2021). Besides, improved SMOTE, auto-encoder-based generative models, conditional generative adversarial network, and GANSO ignore spatial information and temporal information, which limits their classification performance. In addition, our scheme can fit neural networks better and can generate more data samples than previous methods. Therefore, it can reduce over- or under-fitting caused by insufficient data samples in the neural network.

### 4.2. Discriminative brain regions and connections

**Figure 5:**
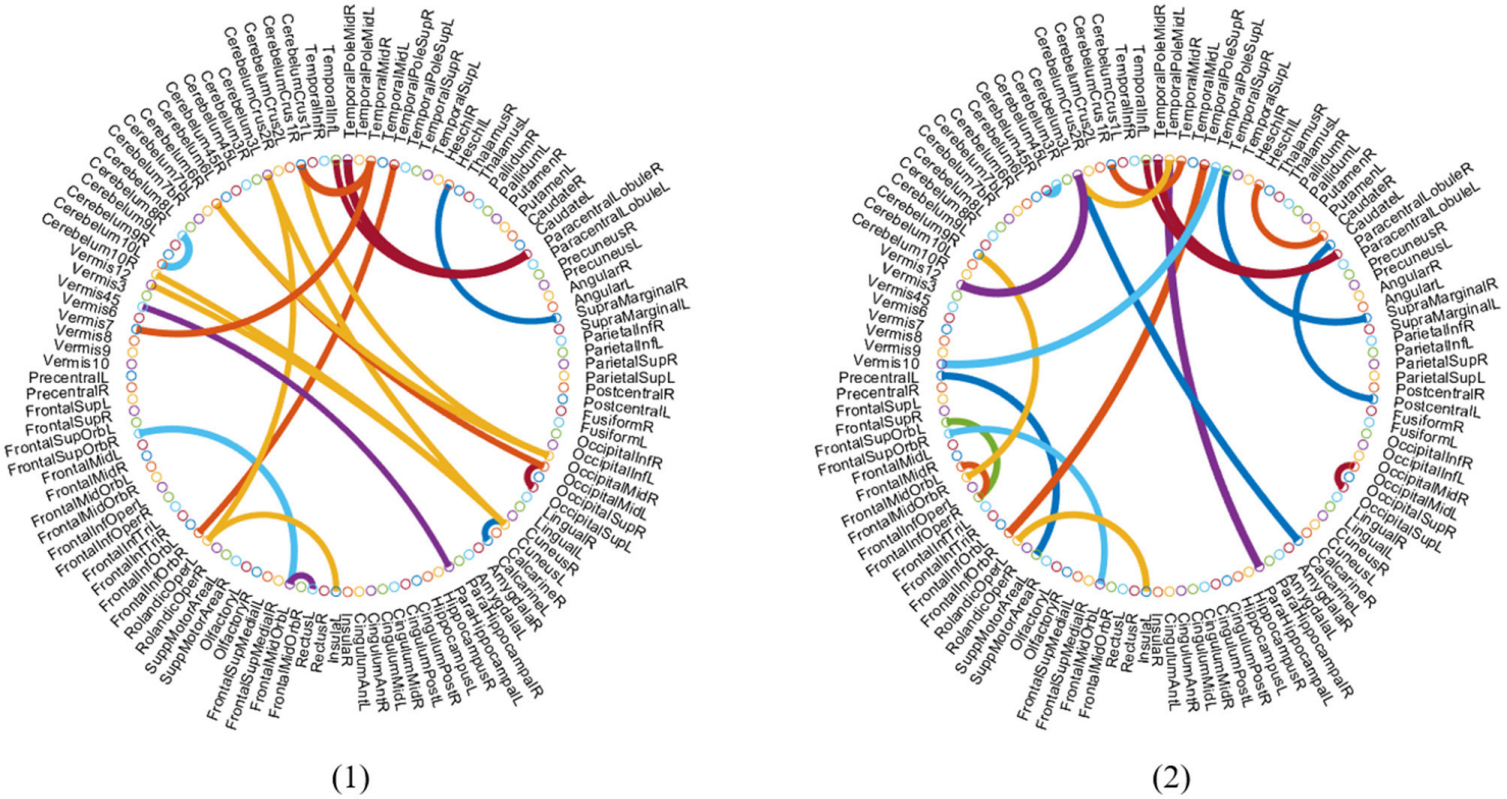
Most discriminative ROIs identified by two classifiers in the task of NC vs. eMCI. (1) Discriminative ROIs based on spatial classifier. (2) Discriminative ROIs based on temporal classifier. The symbol volume of the nodes represents the discriminative power of each brain region.

**Figure 6:**
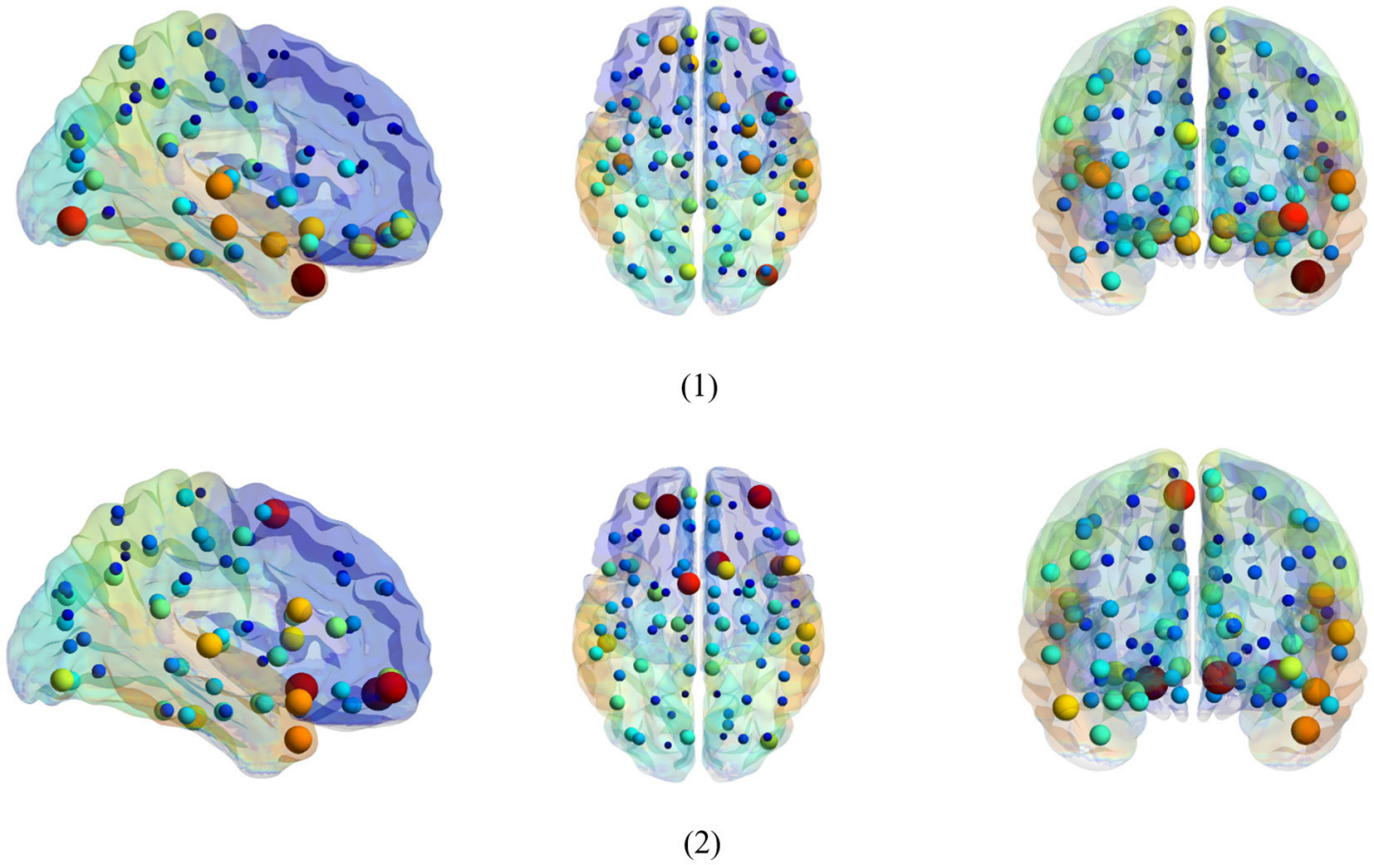
Top 20 discriminant functional connections identified by two classifiers in the task of NC vs. eMCI. (1) Discriminative connections based on spatial classifier. (2) Discriminative connections based on temporal classifier.

It is an essential step to select the discriminative biomarkers in FBN analysis. As shown in Figures 5, 6, we show the most discriminative connections and ROIs based on the weights in two classifiers (i.e., spatial classifier and temporal classifier). Since the weights within each subject can be different, we integrate them and select the most discriminative features based on the weights. We demonstrate the top 20 discriminant connections in Figure 5, where the color of each arc is randomly assigned for better visualization.

Figure 6 is a visualization based on the AAL template, where the symbol volume of the nodes represents the discriminative power of each brain region. We normalize the weights of functional connections obtained using the model, then add the weights of functional connections to the brain regions to obtain the weights discriminating brain regions (Zhang et al., 2021). We can observe that the discriminative ROIs of the brain regions after spatial information augmentation are more concentrated in the right temporal lobe, which corresponds to the fact that the right brain regions of the mentioned Alzheimer's patients are more likely to accelerate aging (Roe et al., 2021). The weights of each brain region after temporal information augmentation are relatively more concentrated in the frontal lobes, similar to previous findings (Agosta et al., 2012).

### 4.3. Effect of different data augmentation degrees

In the proposed STDAC scheme, two data augmentation modules are employed to generate the samples for classification. However, different degrees of data augmentation will lead to different numbers of samples, which may affect the final performance. Therefore, we validate the accuracy of the two data augmentation modules for the classification task of eMCI vs. NC with different degrees.

**Figure 7:**
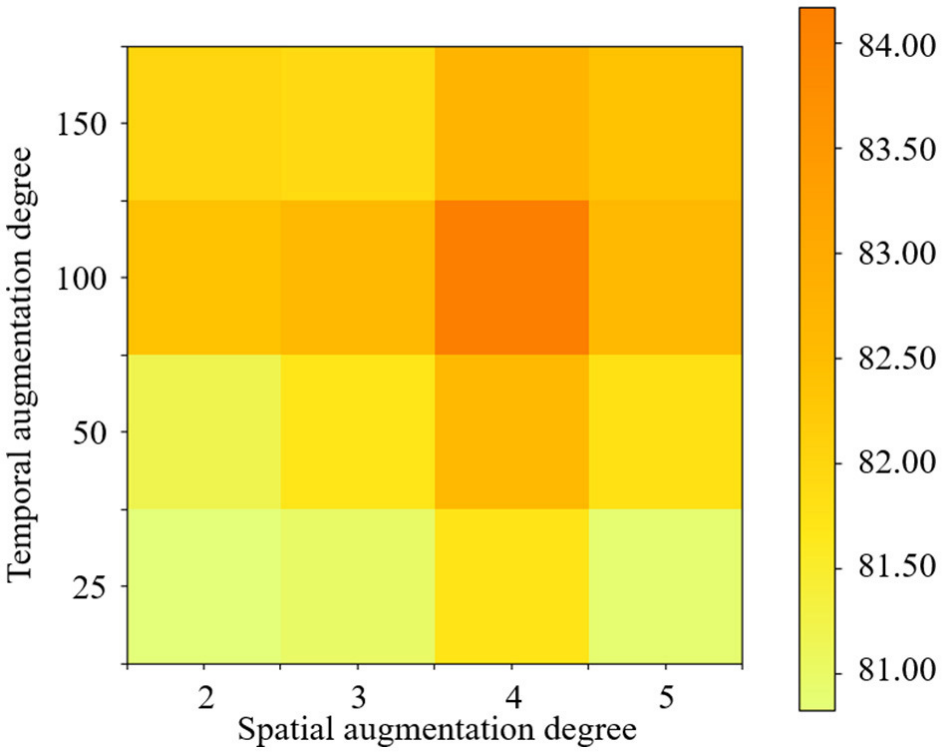
Classification accuracy achieved by the STDAC method using different degrees of spatial and temporal augmentation in the task of NC vs. eMCI.

The experimental results are reported in Figure 7, in which the darkest red on the figures marks the degrees under the best accuracy conditions. It can be observed that the best accuracy is achieved when the spatial degree is 4 and the temporal degree is 100. In addition, whether the degree of data augmentation is too high or too low affects the final result. For example, when the data augmentation degree is higher than 100, the data augmentation effect will also be weakened. The probable reason is that the excessive degree of data augmentation may lead to many duplicate samples. Therefore, we need to select the optimal parameters according to different classification tasks.

### 4.4. Limitations and future work

The proposed STDAC scheme takes both temporal and spatial information of rs-fMRI data into account, and mitigates the occurrence of over- or under-fitting by data augmentation. However, there are several limitations in our present study. *First*, individual differences between different subjects are not fully considered in our scheme, which is an important issue for FBN analysis (Folville et al., 2020; Schabdach et al., 2022) and an important direction for our future improvements. *Besides*, since multi-modal data is taking an increasingly important place in brain analysis (Jia and Lao, 2022; Zhao et al., 2022), the performance of employing one-modal data is limited. Compared with the widely used multi-modal data model (Yu et al., 2021), how to adapt the scheme to the multi-modal data type is a key point in our future work.

## 5. Conclusion

In this study, we perform a spatial-temporal data-augmentation-based classification (STDAC) scheme based on data augmentation through spatial-temporal information for brain disease diagnosis. Specifically, there are three modules in our proposed scheme, including (1) spatial augmentation module based on spatial prior knowledge of brain regions, (2) temporal augmentation module based on random re-sampling of a time series, and (3) spatial-temporal fusion module based on a tensor fusion method to fuse the different information extracted by the previous two modules. Such a technique enables us to alleviate the problem of small sample size while fusing the spatial-temporal information. We evaluate our scheme with the public ADNI and MDD datasets and experimentally demonstrate that the proposed scheme performs well for classification. In addition, we employ different classifiers to verify the robustness of our method. Experiments show that this scheme can be applied to any classifier in different tasks.

## Data availability statement

Publicly available datasets were analyzed in this study. This data can be found at: https://adni.loni.usc.edu/study-design; https://rfmri.org/REST-meta-MDD.

## Ethics statement

Ethical review and approval was not required for the study on human participants in accordance with the local legislation and institutional requirements. The patients/participants provided their written informed consent to participate in this study.

## Author contributions

QL, YZ, and ZW designed the study. QL and YZ downloaded the data, performed the experiments, and drafted the manuscript. LG preprocessed the data and performed some experiments. All authors read and approved the final manuscript.
